# How different defensive formations affect physical match demands in the German Handball-Bundesliga: a comparison of the attacking and defending team

**DOI:** 10.3389/fspor.2026.1811523

**Published:** 2026-06-05

**Authors:** Christian Saal, Patrick Rheinsberg, Christian Baumgart, Joana Brochhagen, Matthias W. Hoppe

**Affiliations:** 1 Health and Physical Activity, Sport Science, Faculty of Humanities, Otto-von-Guericke-Universität Magdeburg, Magdeburg, Germany; 2 Training and Movement Science, Faculty of Sport Science, Leipzig University, Leipzig, Germany; 3 Department of Movement and Training Science, University of Wuppertal, Wuppertal, Germany; 4 Institute of Sport Science and Motology, Philipps University of Marburg, Marburg, Germany

**Keywords:** formation, handball, LPS, physical demands, team tactics

## Abstract

**Introduction:**

In handball, the effects of different defense playing formations are little studied yet, but play a crucial role for managing training volume and intensity when planning specific drills. The aim of the study was to examine how different playing formations within a positional defense phase influence physical match demands for both the attacking and defending team.

**Method:**

A fixed installed local positioning system (Kinexon LPS) was used to collect positional data during the 2023/2024 German Handball-Bundesliga season. The physical match demands were operationalized by the normalized number of accelerations and decelerations (events per 10 min) and the average distance covered (m) by the team within a positional defense phase. The playing formations within a positional defense (6:0, 5:1, 4:2, 3:3) were detected using an existing algorithm that was modified accordingly. In total, 11,863 positional defense phases in equal numbers of players (6 vs. 6) were analyzed including 316 players of 18 clubs. Generalized linear mixed models (GLMMs) were fitted to estimate differences between the playing formations for the attacking and defending team.

**Results:**

Significant three-way interaction effects were observed for deceleration events in defending teams in the 5:1 (0.235 ± 0.063, p<0.001), 4:2 (0.432 ± 0.112, p<0.001), and 3:3 (0.456 ± 0.151, p=0.003) formations. Furthermore, significant differences were found in average distance covered by team for 5:1 (120.0 ± 23.3 m, p<0.001), 4:2 (479.0 ± 45.5 m, p<0.001), 3:3 (776.8 ± 66.0 m, p<0.001) formations compared with 6:0.

**Conclusion:**

We adapted an algorithm to detect positional defense phases (6:0, 5:1, 4:2, and 3:3). With more aggressive playing formations, total distance and numbers of acceleration and deceleration events tended to increase in both the attacking and defending teams. A stronger increase in deceleration events was estimated for defenders in the 3:3 formation, however this finding should be interpreted cautiously due to the low frequency of this formation. Our outcomes may help practitioners manage training intensity and volume when planning specific drills.

## 1 Introduction

Indoor team handball is an intermittent team sport. The playing success is determined by numerous physical, technical, tactical, and psychological factors of the players (1). Thereof, tactical aspects are little studied yet, but play a crucial role for the success and managing training volume and intensity when planning specific drills (2, 3). To accurately investigate tactical aspects in handball, it is crucial to distinguish between phases of ball possession (offense) and non-possession (defense), as the physical constraints may differ significantly (4, 5). When a team possesses the ball, their primary objective is to score a goal, whereas the opposing team’s aim is to prevent this attempt. The transition from non-possession to ball possession triggered by events such as goal scoring, technical fault, offensive foul etc., results in continuous task shifts for both teams. Ball possession phases (BPP; possession/no possession) can be further categorized into transition attack (fast break, TA), positional attack (PA), transition defense (quick retreat, TD), and positional defense (PD) (5). Within these phases, the degree of player organization varies (6). In particular, during PD, teams employ various organizational styles or playing formations, such as a 6:0 formation, where all players align around the 6 m-line, or a 3:3 formation with three players positioned close to the 6 m-line in a first line and the remaining three players positioned further upfield (7). In detail, traditional formations within PD are 6:0, 5:1, 4:2, and 3:3 (5). Of note, defensive tasks can begin before the end of a BPP, ensuring the team is strategically prepared for a rapid transition. More complex defensive formations are also possible, e.g., 3:2:1 or man-to-man.

Modern tracking systems, particularly local positioning systems (LPS), enable the localization of the players and ball with a high accuracy and resolution, which allows the estimation of physical match demands and technical-tactical actions (8). In the 2021/2022 season of the German Handball-Bundesliga, a team’s average total distance covered during a match ranged from 255,501 to 297,801 m (9). At the individual level, however, physical match demands depend strongly on individual playing time, leading to a wide range of values (10–12). Therefore, normalized measures are also used in scientific literature to describe physical match demands (2, 13). According to Michalsik et al. (14) and Fleureau et al. (15), normalized distances (m per 10 min) range from 1,067 to 1,380 m, which is higher compared to other reports with 782 to 896 m (2, 16–19). A handball player performs over 1,100 acceleration and deceleration events exceeding 2 m/$s^{2}$ and falling below $-2\;m/s^{2}$, respectively, per game (20). The majority are low-intensity accelerations and deceleration events, descending in frequency through medium to very high intensities (9). Teams undergo over 50 ball possession phases per game, roughly every 36 s (3). Time spent in offense and defense is relatively similar, with players covering comparable total distances in both roles (14, 19, 21). However, offense may involve slightly higher total distances and high intensity running distances covered, as observed by Manchado et al. (19, 21) and Bassek et al. (4). While these analyses provide important general information about physical match demands in elite handball according to player position and BPP, they do not account for specific offensive and defensive strategies within BPP.

Based on LPS data in handball, methods have been developed to allow for an automated recognition and appropriate evaluation of BPP (7, 22). Current research emphasizes the importance of distinguishing BPP further in PA/PD and TA/TD (7). PA/PD account for approximately 73%–76% of game time, while TA/TD make up 24%–27% (4, 23, 24). PA/PD are more frequent, longer and less intense than TA/TD (4). More normalized distance is covered in TA/TD than in PA/PD, with PA showing higher distance coverage than PD (4, 18). Manchado et al. (25) reported that players performed 0.69 to 1.38 and 0.32 to 0.66 acceleration events per minute in offense and defense, respectively, as well as 0.51 to 1.05 and 0.37 to 0.43 deceleration events per minute (threshold: ±2 m/s^2^). To date, only Guignard et al. (7) have studied the impact of playing formations within PD (6:0, 5:1, 4:2, 3:3) on the physical match demands of a specific handball team in defense. They showed that the physical match demands (normalized sprints, decelerations, normalized distance) of the defending team increases with more aggressive playing formations within PD. The effect of the opposing team’s defensive playing formation within PD on the physical match demands experienced by attackers has not been investigated yet, and therefore more research to clarify the relationships of tactical playing formations and physical match demands in handball is warranted.

The aim of the study was to examine how different playing formations within PD influence physical match demands for both the attacking and defending team. Regarding the results of Guignard et al. (7) we assume that a more aggressive playing formation within PD leads to higher physical match demands in both the attacking and defending team.

## 2 Methods

### 2.1 Design

A retrospective and explorative data analysis was conducted to examine the physical match demands of men’s handball teams competing in the German Handball-Bundesliga during the 2023/2024 season depending on the playing formation within PD. An algorithm was adapted to detect PD of a match with a 6:0, 5:1, 4:2, or 3:3 playing formation based on LPS data (7). Within the phases, the average of the normalized number of acceleration and deceleration events (per 10 min) and average distances covered per team were estimated for the defending and attacking team. The collected positional data was retrieved by an API call provided by Kinexon GmbH with an approval granted by the German Handball-Bundesliga GmbH. Therefore, Ethics Committee clearance was not required. All procedures were conducted in accordance with the Declaration of Helsinki.

### 2.2 Data sample

In total, 269 positional data files were split into first and second half, resulting in 531 half times. No data was available for seven halves. The data includes positional tracking data (x, y coordinates, acceleration in $m/s^{2}$, ball possession ID) for each player (*n* = 316) of 18 clubs and the ball. Only the data of the players on the field that were recorded during the current match were taken into account. The included players can be categorized into tier 3 to 5 according (26).

### 2.3 Data collecting

A fixed installed LPS technology (Kinexon Perform LPS) was used, as described in detail elsewhere (27). Briefly, twelve antennas were positioned around the playing field and connected to a base station. At the same time, all players wore a sensor, which was located between the shoulder blades in a vest under the shirts. The sensor sent time signals using ultra-wide-band technology, which were then transmitted from the antennas to the base station via a wide local area network. This enabled two-dimensional position data at 20 Hz. The coordinate origin (0,0) was centered so that the playing field (40 m × 20 m) was divided into a negative and positive x-half. Additionally, an inertial measurement unit was integrated in the sensor (100 Hz). The data collection was also accompanied by Kinexon employees, who manually marked the start and end of each half time.

### 2.4 Data processing

**Figure 1:**
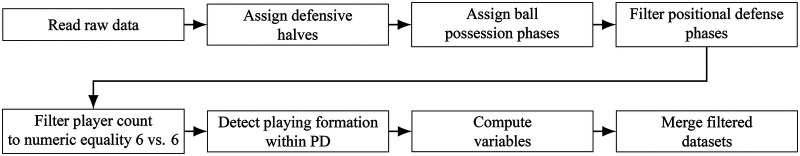
Steps of data processing.

The raw data was proceeded step by step, following a reduction principle, so that only the variables relevant to the PD phase remained; specifically, those derived from equal 6 vs. 6 situations within a defined playing formation within PD (Figure 1). All steps of data processing were done on a High-Performance Computing Cluster at Leipzig University and are described below.

#### 2.4.1 Read raw data

The raw data was cleaned by removing rows containing ball coordinates and missing values, and then sorted by timestamp. Subsequently, any blocks with fewer than 9 rows per timestamp were removed.

#### 2.4.2 Assign defensive halves

The average position of the respective goalkeeper on the x-axis of the pitch was calculated to determine the defensive half. If the average position of a goalkeeper was negative, the negative x-half of the field was assigned to the team as the defensive half, or if the average value was positive, the positive x-half.

#### 2.4.3 Assign ball possession phases

**Figure 2:**
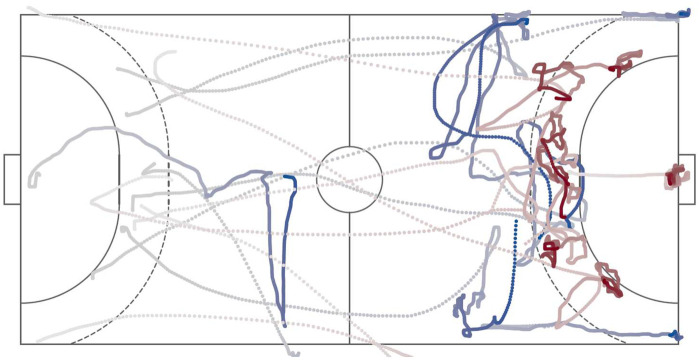
Exemplary representation of a ball possession phase (BPP). The lines represent the running routes of the individual players on the field in the *x* and *y* directions. The attacking team is colored blue and the defending team red. Darker colors indicate that the action is closer to the end of the BPP.

Ball possession IDs were used to assign team ball possession. The BPP were detected based on a set of predefined conditions. Taken into account multiple ball possessions per timestamp, time differences >1 s between blocks (e.g., timeout, wipe pause, missing values), position of players in offensive half, phase duration. BPP with a duration lower than 3 s were removed (Figure 2).

#### 2.4.4 Filter positional defense phases

**Figure 3:**
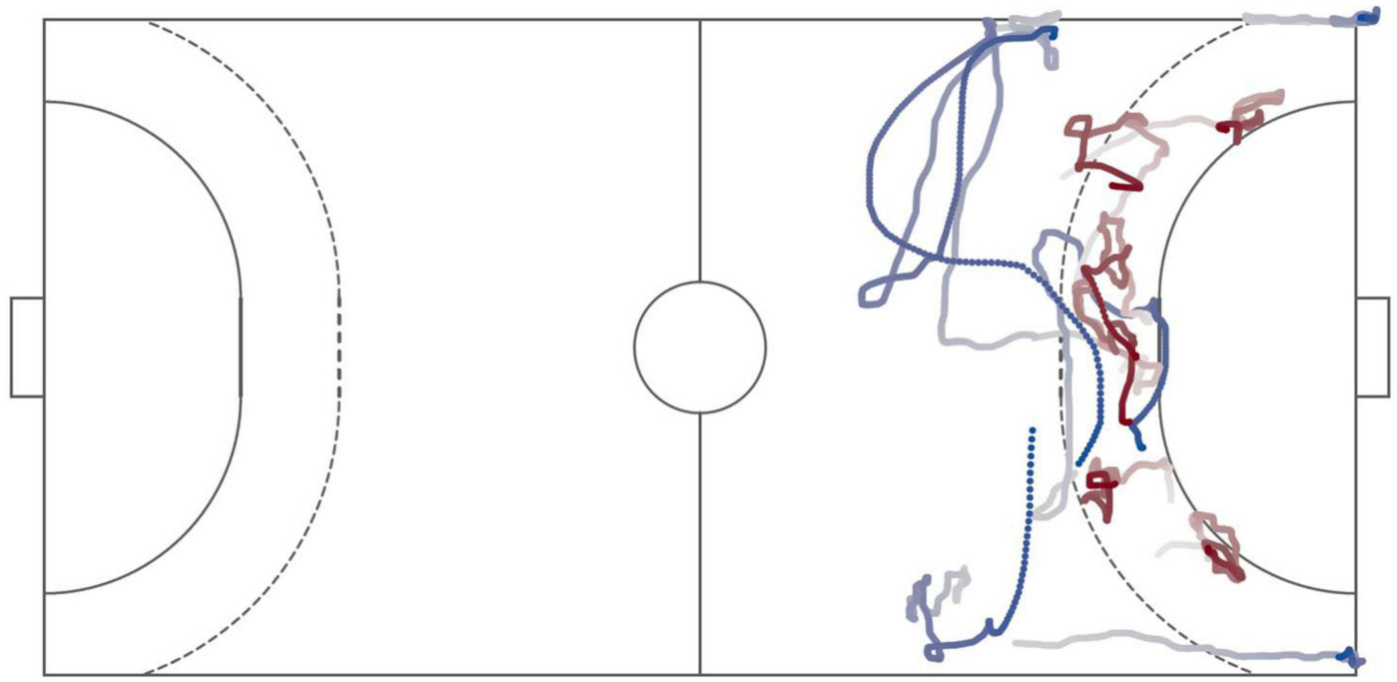
Exemplary representation of a ball possession phase (BPP) after filter PA/PD. The lines represent the running routes of the individual players on the field in the *x* and *y* directions. The attacking team is colored blue and the defending team red. Darker colors indicate that the action is closer to the end of the BPP.

To ensure that TA/TD were not taken into account, predefined conditions were used. Starting condition for PA/PD were the following: all players were located in the attacking half of the team in ball possession and one attacking player was located in the central zone in front of the goal (y=7m, x=11m). Fast defense movements after positional play were cut from the phase if more than 1 player of the attacking team entered the own defense half, ensuring early TD. Only BPP with a duration >12 s were used for further processing. This decision is taken according to the reported value of 6±3 s for a TA/TD phase (4). Figure 3 show a filtered BPP from start to end.

#### 2.4.5 Filter player count to numerical equality 6 vs. 6

Only BPP in which both goalkeeper and all 12 field players were on the field were used.

#### 2.4.6 Detect playing formation within PD

**Figure 4:**
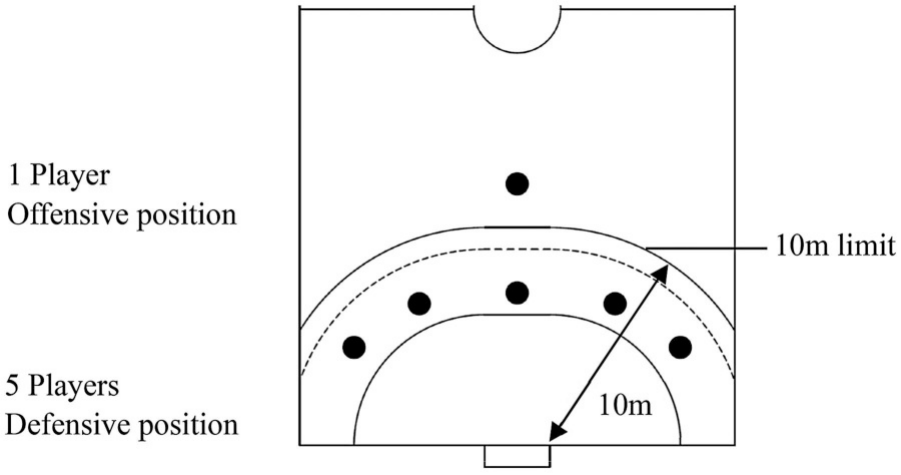
Exemplary representation of a 5:1 defensive formation with a limit of 10 m.

To detect playing formation within PD, we used the approach of calculating the Euclidean distance of each defensive player to the goal line and a limit presented by Guignard et al. (7). This approach was adapted by using two quarter circles and a connecting line parallel to the goal line for defining the limit. After testing several limits (8, 9, 10, and 11 m) the distance of the limit was set to 10 m (Figure 4). The 10 m threshold was chosen on pragmatic and field-specific reasons during algorithm development, as a 9 m threshold led to erroneous non-detection of some 6:0 formations. This likely occurred because laterally anticipatory defenders temporarily positioned themselves outside the 9 m line without representing a genuinely more aggressive defensive formation. The following PD formations were used: 6:0, 5:1, 4:2, and 3:3. The formation that lasted the longest was assigned to the entire PD phase, considering only formations that lasted uninterrupted for more than 2 s. This duration was chosen to ensure that a brief crossing of the threshold would not trigger a formation change.

#### 2.4.7 Compute variables

For each player, the distance covered was calculated using the Euclidean distance between *x* and *y* coordinates, following the approach of Guignard et al. (7) and Lefèvre et al. (18). The average distance covered per team was then summed for each PD phase. For accelerations, the rows in which the threshold for accelerations (positive acceleration 2.0 $m/s^{2}$) was exceeded and for decelerations (negative acceleration −1.5 $m/s^{2}$) was fallen below were identified, according to Kinexons default settings. These asymmetric thresholds were retained because they correspond to the manufacturer’s default settings and deceleration is generally associated with higher mechanical load than acceleration (28, 29). If this condition persisted for at least 0.5 s for a player, an acceleration event was recorded for the PD phase. The total number of such acceleration events across all players of a team was calculated per PD phase. To ensure comparability across PD phases of different duration, all team metrics were normalized to 10 min using $y_{\mathrm{norm}}=(y/t)\times 600$, where y is the raw value and t is the PD phase duration in seconds, following (7, 18). Goalkeepers were excluded from the analysis because their movement profile differs substantially from that of field players. Accordingly, both distance totals and acceleration/deceleration event counts were calculated only for the six field players per team.

### 2.5 Statistical analysis

Data distributions were explored visually and by calculating the quantiles. To analyze differences between the playing formations within PD in acceleration, deceleration events, and distances, two (generalized) linear mixed models (GLMMs) were fitted using the glmmTMB package in R (30, 31). For the distance model, formation (6:0, 5:1, 4:2, 3:3), ball possession (attacking, defending), and their interaction were included as fixed effects (formation * possession), with random intercepts by team (1 | team). For the acceleration/deceleration model, variable (acceleration, deceleration), formation, possession, and their interactions were included as fixed effects (variable * formation * possession), again with random intercepts by team (1 | team). For acceleration and deceleration events, the response variable was modeled using a Tweedie distribution with a log-link function, appropriate for continuous data with a mass at zero and potential overdispersion. Log-transformed values can be converted back to the original scale by exponentiating the estimate using Euler’s number (e). For total average distance per team, given its approximately normal distribution, a gaussian distribution with an identity link function was used. To justify model selection, the final interaction model was compared with simpler candidate models, including a null model and an additive model, using AIC and BIC. To assess model fit and explanatory power, marginal and conditional R^2^ values were calculated for the fitted mixed models. Model fit was further evaluated using simulation-based residual diagnostics from the DHARMa package (32). Post hoc pairwise comparisons of estimated marginal means were conducted using the emmeans package, with Tukey adjustment for multiple comparisons (33).

## 3 Results

In total 11,863 PD phases in equal numbers of players (6 vs. 6) were analyzed. The summed duration of the analyzed phases (PD) averaged 9.2 ± 3.4 min (count: 22.3 ± 7.8) per half time. Proportional 90.3% (*n* = 10,714) are 6:0, 7.1% (*n* = 842) are 5:1, 1.8% (*n* = 209) are 4:2 and 0.8% (*n* = 98) are 3:3 playing formations within PD with a mean duration of 25.1 ± 12.2 s, 21.9 ± 10.0 s, 18.3 ± 8.2 s, and 17.2 ± 6.4 s, respectively. Table 1 shows the descriptive statistics (median and interquartile range) for the number of acceleration and deceleration events and the average distance covered per team for the attacking and defending teams, according to defensive formation.

**Table 1:** Number of phases, location, dispersion for acceleration and deceleration events per 10 min, and mean distance covered per team (m) for attacking and defending teams during numerical equality (6 vs. 6), according to playing formation, within the positional defense phase (PD).

Variable	Defensive formation	*n*	Median	Interquartile range
	6:0	10,714	75.60	82.30
Acceleration attacking	5:1	842	77.40	94.00
(Number of events per 10 min)	4:2	209	91.80	115.40
	3:3	98	80.30	91.70
	6:0	10,714	14.90	43.00
Acceleration defending	5:1	842	23.10	51.70
(Number of events per 10 min)	4:2	209	29.20	56.60
	3:3	98	40.40	75.70
	6:0	10,714	80.90	63.70
Deceleration attacking	5:1	842	77.90	64.90
(Number of events per 10 min)	4:2	209	88.30	75.70
	3:3	98	97.60	85.20
	6:0	10,714	23.80	45.30
Deceleration defending	5:1	842	34.00	60.30
(Number of events per 10 min)	4:2	209	48.90	89.90
	3:3	98	69.60	78.90
	6:0	10,714	4,302.60	925.30
Average distance covered per team	5:1	842	4,422.10	1,011.20
attacking (m)	4:2	209	4,683.40	1,172.70
	3:3	98	5,042.70	1,288.00
	6:0	10,714	2,877.80	599.40
Average distance covered per team	5:1	842	3,124.10	704.60
defending (m)	4:2	209	3,460.70	834.80
	3:3	98	3,944.40	865.10

### 3.1 Acceleration and deceleration

**Figure 5:**
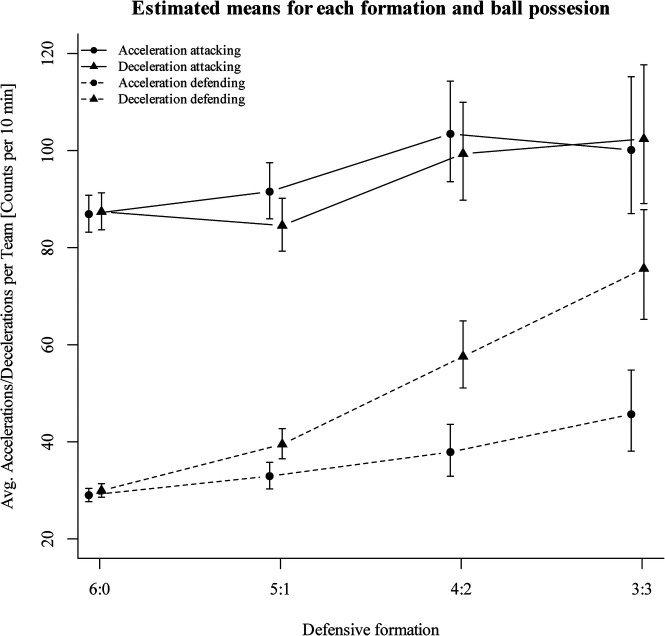
Estimated model-based means of the number of average acceleration and deceleration events per team (per 10 min) for the attacking and defending team during positional defense phases (PD) in numerical equality (6 vs. 6), by playing formation. Estimates are shown on the response scale. Error bars indicate 95% confidence intervals.

Table 2 presents the fixed effects and Figure 5 the estimated means for each playing formation within PD, team in possession (attacking, defending), and variable (accelerations, decelerations). For this model, the marginal and conditional R^2^ values were 0.345 and 0.354, respectively. Simulation-based residual diagnostics using DHARMa showed no evidence of zero inflation (p = 0.688) or dispersion (p = 0.384), but significant deviations from uniformity (p<0.001), outlier frequency (p<0.001), and quantile expectations (p<0.001). Compared with the null model (AIC = 422,077, BIC = 422,112) and the additive model (AIC = 407,613, BIC = 407,692), the final interaction model showed the lowest AIC and BIC values (AIC = 407,456, BIC = 407,623). Likelihood-ratio tests also supported the interaction model over the simpler models (both p<0.001). The intercept of 4.465 is the estimated log-value for the reference condition (acceleration, 6:0, attacking team). For attacking teams, significant differences in acceleration counts compared to the 6:0 formation were found for 5:1 (0.052 ± 0.025, p = 0.040, 5.3%), 4:2 (0.174 ± 0.047, p<0.001, 19%), and 3:3 (0.141 ± 0.069, p = 0.040, 15.1%). For the defending team, the number of acceleration and deceleration events is significantly lower by −66.6% (−1.097 ± 0.013, p<0.001) in the reference condition. Additional two-way interactions indicated that the differences between acceleration and deceleration events and between formations in defending teams vary across specific formations (e.g., deceleration × formation 5:1, 4:2, 3:3, and formation × defending terms; see Table 2). Significant three-way interaction effects (variable × formation × possession) were observed for deceleration events in defending teams in the 5:1 (0.235 ± 0.063, p<0.001), 4:2 (0.432 ± 0.112, p<0.001), and 3:3 (0.456 ± 0.151, p = 0.003) formations, indicating that differences between acceleration and deceleration events depend on both defensive formation and team possession. A post hoc comparison showed that, for deceleration in the defending team, the estimated mean was higher in the 3:3 than in the 6:0 formation (75.7 vs. 30.0 events per 10 min; ratio = 2.53, 95% CI: 2.09–3.06, p<0.001).

**Table 2:** Fixed effects from the GLMM predicting the average number of acceleration and deceleration events per team during positional defense phases (PD), reported on the log-scale.

Predictor	Estimate	SE	z	*p*
(Intercept)	4.465	0.022	200.62	<.001***
Deceleration	0.006	0.010	0.57	n.s.
Formation 5:1	0.052	0.025	2.05	<.05*
Formation 4:2	0.174	0.047	3.71	<.001***
Formation 3:3	0.141	0.069	2.06	<.05*
Defending	−1.097	0.013	−83.43	<.001***
Deceleration × Formation 5:1	−0.085	0.036	−2.34	<.05*
Deceleration × Formation 4:2	−0.046	0.067	−0.69	n.s.
Deceleration × Formation 3:3	0.017	0.097	0.17	n.s.
Deceleration × Defending	0.026	0.018	1.43	n.s.
Formation 5:1 × Defending	0.075	0.046	1.63	n.s.
Formation 4:2 × Defending	0.093	0.084	1.11	n.s.
Formation 3:3 × Defending	0.312	0.114	2.74	<.01**
Deceleration × Formation 5:1 × Defending	0.235	0.063	3.69	<.001***
Deceleration × Formation 4:2 × Defending	0.432	0.112	3.85	<.001***
Deceleration × Formation 3:3 × Defending	0.456	0.151	3.01	<.01**

Reference categories are variable = acceleration, formation = 6:0, and possession = attacking. *p<.05, **p<.01, *** p<.001.

### 3.2 Average distance covered per team

**Figure 6:**
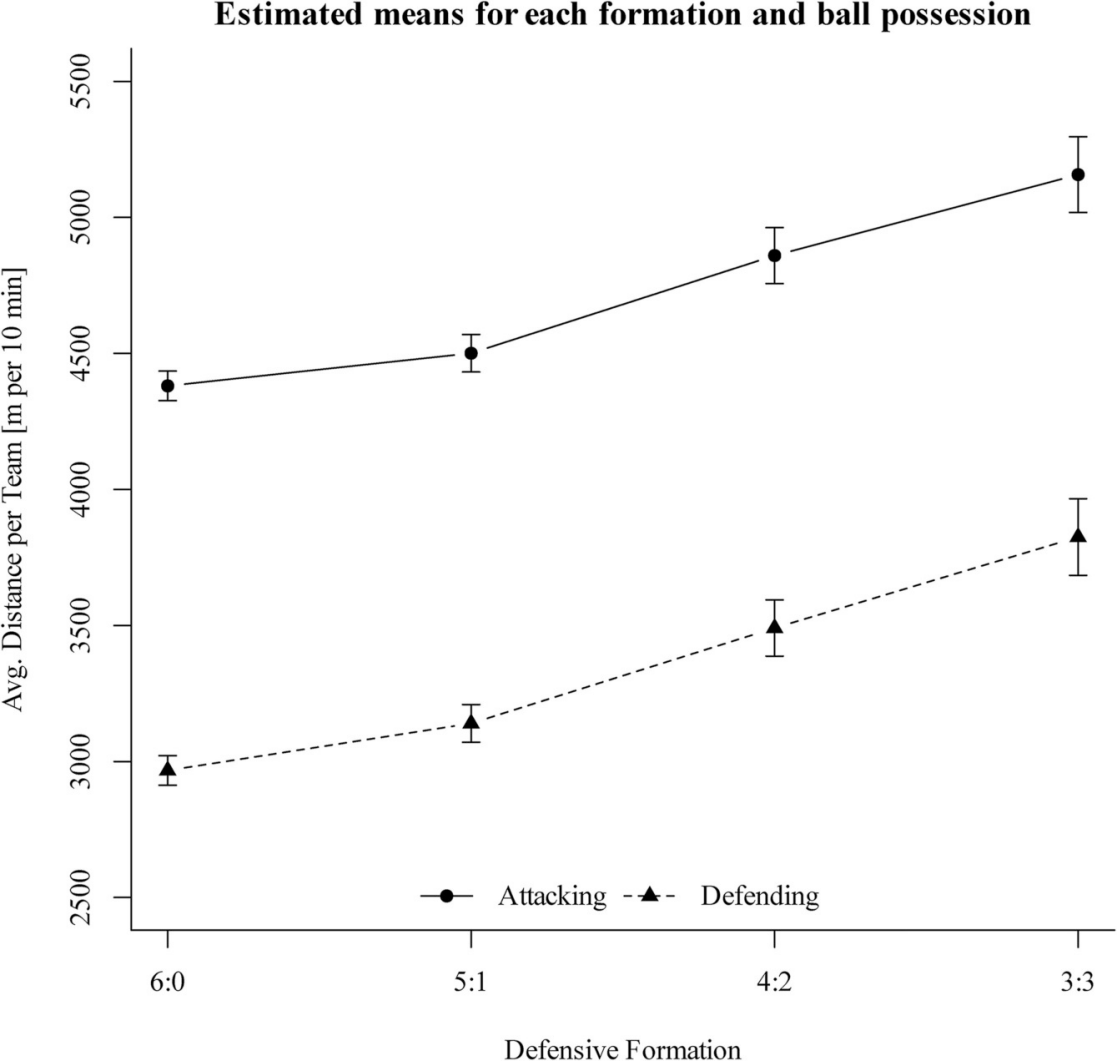
Estimated model-based means of the average distance per team (per 10 min) for the attacking and defending team during positional defense phases (PD) in numerical equality (6 vs. 6), by playing formation. Estimates are shown on the response scale. Error bars indicate 95% confidence intervals.

Table 3 shows the fixed effects and Figure 6 the estimated means for each playing formation within PD, team in possession (attacking, defending). For this model, the marginal and conditional R^2^ values were 0.537 and 0.551, respectively. Simulation-based residual diagnostics using DHARMa showed no evidence of dispersion (p = 0.896). Significant deviations from uniformity (p<0.001) and outlier frequency (p<0.001) were observed. Compared with the null model (AIC = 393,355, BIC = 393,379), the final interaction model showed substantially lower AIC and BIC values (AIC = 374,840, BIC = 374,921). Because the additive mixed model did not converge, likelihood-ratio comparisons with this model were not performed. The intercept of 4,381 m is the estimated value for the reference (6:0, attacking team). Significant differences were found for 5:1 (120 ± 23.35 m, p<0.001, 2.7%), 4:2 (479 ± 45.50 m, p<0.001, 10.9%), 3:3 (777 ± 66.10 m, p<0.001, 17.7%) against 6:0 in attacking teams. For the defending team, the average distance covered per team was significantly lower by 1,413 ± 8.96 m (p<0.001), corresponding to about 32.3% less than for the attacking team in the reference condition. Significant interaction effects were not found. A post hoc comparison showed that, for the defending team, the estimated mean distance covered was higher in the 3:3 than in the 6:0 formation (3,825 vs. 2,967 m per 10 min; difference = 858 m, 95% CI: 685.7–1,030 m, p<0.001).

**Table 3:** Fixed effects from the GLMM predicting the average distance covered per team during positional defense phases (PD), reported on the response scale.

Predictor	Estimate	SE	z	*p*
(Intercept)	4,381	27.72	158.03	<.001***
Formation 5-1	120	23.35	5.14	<.001***
Formation 4-2	479	45.50	10.53	<.001***
Formation 3-3	777	66.10	11.75	<.001***
Defending	−1,413	8.96	−157.76	<.001***
5-1 × Defending	53	33.40	1.58	n.s.
4-2 × Defending	44	64.72	0.69	n.s.
3-3 × Defending	81	94.18	0.86	n.s.

Reference categories are formation: 6:0, possession: attacking. p<.05 *, p<.01 **, p<.001 ***.

## 4 Discussion

The aim of the study was to examine how different playing formations within a positional defense phase influence physical match demands for both the attacking and defending team. Our main findings show: (1) The number of acceleration and deceleration events was 67% lower in the defending team compared to the attacking team, and increased with a more aggressive playing formation within PD. Additionally, this playing formation effect was more pronounced for deceleration events in the defending team. (2) The normalized average distance covered per team was lower in the defending team than in the attacking team and increased with a more aggressive playing formation within PD.

The first main finding was, that the number of acceleration and deceleration events per 10 min were lower by 67% in the defending team compared to the attacking team (Figure 5). This finding aligns with another study, which showed that the attacking team performed more acceleration and deceleration events per 10 min than the defending team (25). The reported values are comparable considering the different threshold used for deceleration (in our study −1.5m/s). We additionally found an increase of the number of acceleration and deceleration events in the defending and attacking team with more aggressive playing formation within PD. Also, this finding underlines the results from a study including the condition formation (6:0, 5:1, 4:2, and 3:3), which also showed an increasing number of deceleration events for the defending team (7). However, the authors reported deceleration counts per 10 min for the defending team that were approximately ten times higher across formations (6:0, *n* = 1,225; 5:1, *n* = 1,395; 4:2, *n* = 1,529; 3:3, *n* = 1,633). This may be due to the different threshold values. In our study, it was set to 2.0 $m/s^{2}$ for accelerations and −1.5 $m/s^{2}$ for decelerations and had to last for 0.5 s. In contrast, the threshold in the forementioned study was −0.2 $m/s^{2}$, which could be exceeded much more quickly. For the detection of the different formations, we followed the approach of Guignard et al. (7) except that we shift the border for detecting team formations from 9 to 10 m. This led to the fact that offensively anticipating and receiving the attackers in front of the 9 m area did not result in the detection of a more aggressive playing formation. This could explain the different reported proportions of detected defensive playing formation in our study (6:0: 90%, 5:1: 7%, 4:2: 2%, 3:3: 1%) compared to the mentioned study (6:0: 20%, 5:1: 50%, 4:2: 30%, 3:3: 15%). In our study, we also included the attacking team and found that the effect on the number of deceleration events was more pronounced in the defending team than in the attacking team, which is a novel finding. A *post-hoc* comparison showed that, for deceleration events in the defending team, the estimated mean was higher in 3:3 than in 6:0 (75.7 vs. 30.0 events per 10 min; 152.3%). However, this result should be interpreted with caution because of the very low frequency of the 3:3 formation. Taken together, methodological differences limit the comparability of the reported values. Nevertheless, our findings suggest that the number of acceleration and deceleration events was lower in the defending team and tended to increase with more aggressive playing formations during PD.

The second main finding showed that normalized average distance covered per team was lower in the defending team than in the attacking team and tended to increase with more aggressive playing formations within PD in both groups (Figure 6). Our reported normalized values of average distance covered per team of 4,300–5,000 m for the attacking team and 2,900–3,900 m for the defending team are comparable with those reported in other studies (9, 18, 20). Also, the significant difference between average distance covered and locomotion activities (e.g., walking, jogging) between the attacking and defending team were reported in other studies (4, 19, 21). However, the normalized average distance covered by the defending team is lower than the values reported in another study (7). Nevertheless, an increase in running distance with a more aggressive team formation within PD was also observed (7). The here observed findings in terms of the playing volume align with those discussed for playing intensity before and support our methodological approaches. However, further research using more comprehensive insights into the intensity domain regarding the impact of tactical aspects in team handball is needed.

In summary, the physical match demands tended to increase with more aggressive playing formations within PD in both the attacking and defending team. In particular, the number of deceleration events appeared higher in the defending team in the 3:3 playing formation. However, this finding should be interpreted cautiously because of the very low frequency of this formation. One explanation for the increase in physical demands with more aggressive playing formations may be found in the spatial and organizational aspect of the different playing formations within PD. Aggressive playing formations (e.g., 3:3) within PD may enlarge the spaces that must be defended and may therefore be associated with tighter marking, more off-ball movement, greater distances covered, and more accelerations and decelerations and changes of direction by both attackers and defenders. In more aggressive playing formations within PD, a higher proportion of unstructured attacks may be observed, whereas more defensive playing formations (e.g., 6:0) could be associated with an increased proportion of structured attacks (6). In contrast, in a 3:3 playing formation, defenders may need to react more often to attackers’ cuts, feints and direction changes, which may be associated with the higher number of deceleration events observed in the defending team. These findings may help coaches in managing training intensity and volume when planning game-related competition drills.

## 5 Limitations

While our study clearly increased the knowledge of the relationships between tactical, technical and physical aspects in German Bundesliga team handball players, few limitations should be mentioned. First, several specific assumptions were applied in an attempt to capture PD. However, these assumptions and their thresholds were defined visually, which led to occasional discrepancies, for example, when movements at the end of an episode already belonged to the subsequent phase, reflecting the general difficulty of precisely determining the start and end of each offensive phase. Second, special situations such as free throws and penalties could not be explicitly removed by the algorithm and were partly included in the data. Possession phases were only interrupted for temporal reasons (>1 s stoppage or ≥2 s unassigned ball possession), so other interruptions (e.g., fouls, passive play) were not systematically excluded. Third, this study was restricted to four formations (6:0, 5:1, 4:2, 3:3), while more complex playing formations (e.g., 3:2:1, man-to-man) were not considered. Fourth, acceleration and deceleration events were identified using asymmetric thresholds (2.0 vs. $-1.5\;m/s^{2}$), following the manufacturer’s default settings. This approach reflects the higher mechanical load typically associated with decelerations, but it may affect the direct comparability of acceleration and deceleration counts. Sensitivity analyses using alternative thresholds were not performed and should be considered in future research. The approach classifies formations only by the number of players positioned offensively or defensively (10 m threshold), so specific defensive systems may be misclassified or merged (e.g., 3:2:1 within 5:1, 4:2 or 3:3). Furthermore, the algorithm classified formations based on sustained threshold crossings relative to the goal line and may therefore not fully capture edge cases such as laterally displaced defenders or vertically staggered defensive structures that remain below the predefined threshold. In addition, the modified formation-detection algorithm was not formally validated against manual coding based on synchronized video data. Such a validation would require multiple raters and agreement analyses and was beyond the scope of the present study. Fifth, the analyses were conducted at the team level and did not account for player positions. Acceleration, deceleration, and total distance profiles in handball are highly position-specific (2, 13). It remains unclear whether the position-specific patterns follow the same trends as those observed at the team level. Future studies should therefore incorporate positional information, for example in position-specific analyses or as an additional fixed or random effect. These limitations offer possibilities for future research within the largely unexplored field of the interplay between tactical, technical, and physical aspects in team sports such as handball.

## 6 Conclusion

We adapted an existing algorithm to detect positional defense phases of a handball match with a 6:0 ,5:1, 4:2, or 3:3 defensive playing formation based on LPS data from the German Handball-Bundesliga. We found that the number of acceleration and deceleration events and the total distance tended to increase with more aggressive playing formations within PD for both teams, attacking and defending. Furthermore, the increase of the numbers of deceleration is much higher in the defending team in a 3:3 formation compared to the attacking team. Our outcomes can help practitioners managing training intensity and volume and, when planning specific drills.

## Data Availability

The data analyzed in this study is subject to the following licenses/restrictions: The collected data is freely available for each team competing in the German Handball-Bundesliga. Requests to access these datasets should be directed to info@daikin-hbl.de.

## References

[1] Wagner H Finkenzeller T Würth S von Duvillard SP. Individual and team performance in team-handball: a review. J Sports Sci Med. (2014) 13:808–16. 25435773 PMC4234950

[2] García-Sánchez C Navarro RM Karcher C. Physical demands during official competitions in elite handball: a systematic review. Int J Environ Res Public Health. (2023) 20:2–30. 10.3390/ijerph20043353 PMC9965087 36834047

[3] Karcher C Buchheit M. On-court demands of elite handball, with special reference to playing positions. Sports Med. (2014) 44:797–814. 10.1007/s40279-014-0164-z 24682948

[4] Bassek M Raabe D Banning A Memmert D Rein R. Analysis of contextualized intensity in men’s elite handball using graph-based deep learning. J Sports Sci. (2023) 41:1299–308. 10.1080/02640414.2023.2268366 37850373

[5] Hapková I Estriga L Rot C. Teaching Handball. Volume 1 Teacher Guidelines. Cairo, Egypt: Police Press (2019). Available online at: https://www.ihf.info/sites/default/files/2021-06/H@SBooklet_1.pdf (Accessed July 03, 2025).

[6] Lozano D Camerino O. Effectiveness of offensive systems in handball. Apunts Educ Fís Deportes. (2012) 108:70–81. 10.5672/apunts.2014-0983.es.(2012/2).108.08

[7] Guignard B Karcher C Reche X Font R Komar J. Contextualizing physical data in professional handball: using local positioning systems to automatically define defensive organizations. Sensors. (2022) 22:5692. 10.3390/s22155692 35957247 PMC9370953

[8] Blauberger P Marzilger R Lames M. Validation of player and ball tracking with a local positioning system. Sensors. (2021) 21:1465. 10.3390/s21041465 33672459 PMC7923412

[9] Saal C Baumgart C Wegener F Ackermann N Sölter F Hoppe MW. Physical match demands of four LIQUI-MOLY Handball-Bundesliga teams from 2019–2022: effects of season, team, match outcome, playing position, and halftime. Front Sports Act Liv. (2023) 5:1183881. 10.3389/fspor.2023.1183881 PMC10246450 37293438

[10] Póvoas SCA Ascensão AAMR Magalhães J Seabra AF Krustrup P Soares JMC, et al. Physiological demands of elite team handball with special reference to playing position. J Strength Cond Res. (2014) 28:430–42. 10.1519/JSC.0b013e3182a953b1 24473468

[11] Póvoas SCA Ascensão AAMR Magalhães J Seabra AFT Krustrup P Soares JMC, et al. Analysis of fatigue development during elite male handball matches. J Strength Cond Res. (2014) 28:2640–8. 10.1519/JSC.0000000000000424 24552799

[12] Póvoas SCA Seabra AFT Ascensão AAMR Magalhães J Soares JMC Rebelo ANC. Physical and physiological demands of elite team handball. J Strength Cond Res. (2012) 26:3365–75. 10.1519/JSC.0b013e318248aeee 22222325

[13] Randers MB Póvoas S. Positional differences in match performance for key elite male team handball players at the EHF EURO 2022. Int J Perform Anal Sport. (2026) 1–17. 10.1080/24748668.2026.2654334

[14] Michalsik LB Aagaard P Madsen K. Locomotion characteristics and match-induced impairments in physical performance in male elite team handball players. Int J Sports Med. (2013) 34:590–9. 10.1055/s-0032-1329989 23258606

[15] Fleureau A Rabita G Leduc C Buchheit M Lacome M. Peak locomotor intensity in elite handball players: a first insight into player position differences and training practices. J Strength Cond Res. (2022) 37:432–8. 10.1519/JSC.0000000000004247 36026458

[16] Büchel D Jakobsmeyer R Döring M Adams M Rückert U Baumeister J. Effect of playing position and time on-court on activity profiles in german elite team handball. Int J Perform Anal Sport. (2019) 19:832–44. 10.1080/24748668.2019.1663071

[17] Cardinale M Whiteley R Hosny AA Popovic N. Activity profiles and positional differences of handball players during the world championships in Qatar 2015. Int J Sports Physiol Perform. (2017) 12:908–15. 10.1123/ijspp.2016-0314 27918655

[18] Lefèvre T Guignard B Karcher C Reche X Font R Komar J. A deep dive into the use of local positioning system in professional handball: automatic detection of players’ orientation, position and game phases to analyse specific physical demands. PLoS One. (2023) 18:e0289752. 10.1371/journal.pone.0289752 37585452 PMC10431627

[19] Manchado C Pueo B Chirosa-Rios LJ Tortosa-Martínez J. Time-motion analysis by playing positions of male handball players during the european championship 2020. Int J Environ Res Public Health. (2021) 18:2787. 10.3390/ijerph18062787 33801814 PMC8002104

[20] Font R Karcher C Reche X Carmona G Tremps V Irurtia A. Monitoring external load in elite male handball players depending on playing positions. Biol Sport. (2021) 38:475–81. 10.5114/biolsport.2021.101123 34475629 PMC8329973

[21] Manchado C Tortosa Martínez J Pueo B Cortell Tormo JM Vila H Ferragut C, et al. High-performance handball player’s time-motion analysis by playing positions. Int J Environ Res Public Health. (2020) 17:6768. 10.3390/ijerph17186768 32957441 PMC7559068

[22] Bassek M Memmert D Rein R. Automatic formation recognition in handball using template matching. In: Zhang H, Lames M, Baca A, Wu Y, editors. *Proceedings of the 14th International Symposium on Computer Science in Sport (IACSS 2023)*. Singapore: Springer Nature Singapore (2024). p. 10–17.

[23] Ferrari W Sarmento H Marques A Dias G Sousa T Sánchez-Miguel PA, et al. Influence of tactical and situational variables on offensive sequences during elite European handball matches. Front Psychol. (2022) 13:861263. 10.3389/fpsyg.2022.861263 35783727 PMC9249053

[24] Rogulj N Vuleta D Milanović D Čavala M Foretic N. The efficiency of elements of collective attack tactics in handball. Kinesiol Slov. (2011) 17:5–14.

[25] Manchado C Tortosa-Martinez J Marcos-Jorquera D Gilart-Iglesias V Pueo B Chirosa-Rios L. Monitoring external load during real competition in male handball players through big data analytics: differences by playing positions. Kinesiology. (2024) 56:12–23. 10.26582/k.56.1.2

[26] McKay AKA Stellingwerff T Smith ES Martin DT Mujika I Goosey-Tolfrey VL, et al. Defining training and performance caliber: a participant classification framework. Int J Sports Physiol Perform. (2022) 17:317–31. 10.1123/ijspp.2021-0451 34965513

[27] Hoppe MW Baumgart C Polglaze T Freiwald J. Validity and reliability of GPS and LPS for measuring distances covered and sprint mechanical properties in team sports. PLoS One. (2018) 13:e0192708. 10.1371/journal.pone.0192708 29420620 PMC5805339

[28] Harper DJ Carling C Kiely J. High-intensity acceleration and deceleration demands in elite team sports competitive match play: a systematic review and meta-analysis of observational studies. Sports Med. (2019) 49:1923–47. 10.1007/s40279-019-01170-1 31506901 PMC6851047

[29] McBurnie AJ Harper DJ Jones PA Dos’Santos T. Deceleration training in team sports: another potential ‘vaccine’ for sports-related injury? Sports Med. (2022) 52:1–12. 10.1007/s40279-021-01583-x 34716561 PMC8761154

[30] Brooks ME Kristensen K van Benthem KJ Magnusson A Berg CW Nielsen A, et al. glmmTMB balances speed and flexibility among packages for zero-inflated generalized linear mixed modeling. R J. (2017) 9:378–400. 10.32614/RJ-2017-066

[31] McGillycuddy M Warton DI Popovic G Bolker BM. Parsimoniously fitting large multivariate random effects in glmmTMB. J Stat Softw. (2025) 112:1–19. 10.18637/jss.v112.i01

[32] Hartig F. DHARMa: residual diagnostics for hierarchical (multi-level/mixed) regression models. *R package version 0.4.7* (2024). 10.32614/CRAN.package.DHARMa

[33] Lenth RV. emmeans: estimated marginal means, aka least-squares means. *R package version 1.11.1* (2025). 10.32614/CRAN.package.emmeans

